# Editorial: Physiology of invertebrate sensing in extreme conditions and hostile environment

**DOI:** 10.3389/fphys.2023.1334716

**Published:** 2023-11-20

**Authors:** Michele Cesari, Chiara Boschetti, Thomas C. Boothby

**Affiliations:** ^1^ Department of Life Sciences, University of Modena and Reggio Emilia, Modena, Italy; ^2^ School of Biological and Marine Sciences, University of Plymouth, Plymouth, United Kingdom; ^3^ Department of Molecular Biology, University of Wyoming, Laramie, WY, United States

**Keywords:** extreme environments, stress tolerance, cryptobiosis, adaptation, invertebrates

## Introduction

Habitats with considerably harsh conditions such as cold or hot temperatures, accessibility to water and to different energy sources, or under high atmospheric pressure, are very hard to survive in and are thus considered extreme. Examples of extreme environments include the geographical poles, arid deserts, volcanoes, deep ocean trenches, the upper atmosphere, and outer space. Even within apparently non-extreme environments, there are “pockets” of unsuitable environments, and some invertebrate species can survive and in some cases thrive in such difficult conditions, presenting specific adaptations that are usually a result of long-term evolution (Rebecchi et al., 2007). Now, in an age of climate change and anthropogenic pressure, organisms face warmer, more acidic, more toxic, and in general more severe environments than ever before. These variations are sometimes sudden, in some cases overcoming the resistance ability of individual invertebrate species, thus altering the level of resilience, and therefore composition, of entire communities and ecosystems.

The aim of this Research Topic is to provide a major interdisciplinary synthesis of recent advances in the study on invertebrate strategies and responses to stress under unfavorable conditions. Understanding the mechanisms that support such an extreme stress response may help in climate change management and mitigation, and biodiversity conservation, and may also prove invaluable for future potential biotechnological applications that support food security, conservation, and disease prevention/treatment.

There are four original research articles and one mini-review published in this Research Topic, covering tardigrades and bumble bees. These contributions provide an engaging overview of different aspects of survival responses to extreme conditions and hostile environments.

Hagelbäck and Jönsson present a study on tolerance to low oxygen conditions (hypoxia) in tardigrades, in particular evaluating the response to exposure to hypoxia for time periods up to 24 h in two different species of tardigrades, *Richtersius* cf. *coronifer* and *H. exemplaris*. Survival was high in both species after the shortest exposures to hypoxia. However, it declined with longer exposures, with almost complete failure to recover after 24 h. Both species were able to recover after exposure to severe hypoxia, but only if exposure was for relatively short periods. *R*. cf. *coronifer* tended to be more tolerant than *H. exemplaris*, indicating that tardigrade species have different sensitivity and response patterns to exposure to hypoxia.

Kumar Nagwani et al. report on how the hypomagnetic field affects a limno-terrestrial tardigrade species, *Paramacrobiotus experimentalis*. After hypomagnetic field treatments of different durations, tardigrade survival rate and mitochondrial inner membrane potential level were scored, taking also into account the relationship between the age and sex of the tested specimens. Active *P. experimentalis* individuals displayed a high survival rate, although males appeared to be more sensitive to the hypomagnetic field. Age- and sex-related differences were found in mitochondrial inner membrane potential levels, which were also dependent on hypomagnetic field duration. The data reported are an interesting contribution to the understanding of tardigrade aging and their resistance to extreme conditions, which in turn may be useful for future space explorations.

Giovannini et al. delineate the effect of increasing temperature on Antarctic terrestrial communities. In particular, the tardigrade *A. antarcticus* was used as a model animal to gain insights on its life history traits and fitness by rearing specimens at two different and increasing temperatures (5°C vs. 15°C). Moreover, the authors present the first transcriptome analysis of *A. antarcticus*, analyzing adult animals exposed to a gradual increase of temperature to find differentially expressed genes under short and long-term heat stress. *A. antarcticus* specimens reared at 5°C lived longer reach sexual maturity later, laid more eggs (which hatched in longer time and in lower percentage) compared with animals reared at 15°C. The short-term heat exposure led to significant changes at transcriptomic level, suggesting alterations of mitochondrial activity and oxido-reductive processes, whereas the long-term exposure was more limited, probably indicating an acclimation response, which could allow *A. antarcticus* to cope with increasing temperatures imposed by global climate change.

Hvidepil and Møbjerg detail the osmotic and chemical stress tolerance in the marine tidal tardigrade *Echiniscoides sigismundi*. This heterotardigrade is capable to enter the tun state following exposure to saturated seawater and upon exposure to locality seawater containing the mitochondrial uncoupler 2,4-Dinitrophenol, providing evidence of osmobiosis and chemobiosis, *i.e*., cryptobiosis induced by high levels of osmolytes and toxicants, respectively. However, small decreases in survival were observed following simultaneous exposure to both stresses, indicating that the tardigrades may not be entirely ametabolic while in the osmobiotic tun. The authors discussed their data in relation to other studies on cryptobiosis and consider the fact that the mechanisms underlying osmobiosis and anhydrobiosis are overlapping, suggesting that osmobiosis may represent the evolutionary forerunner of cryptobiosis.

White and Dillon describe how temperatures below heat tolerance limits can affect organisms. Using bumble bees (genus *Bombus*) as a case study, they outline the effects of heat stresses below the critical thermal maximum on mortality, behavior, morphology and fertility of these important pollinators. This mini-review suggests that predicting climate change impacts for diverse invertebrates will require a more nuanced understanding of the effects of heat exposure, such as additional studies of carry-over effects and compensatory responses by colonies.

## Conclusion

Overall, this Research Topic provides original research data and an in-depth review that enlighten us on responses by invertebrates to different extreme stressors and environments. Additionally, we anticipate that findings from these studies will pave the way for more investigations, as well as help leverage the potential of the field of invertebrate biology in supporting evidence-based policymaking in climate change management and mitigation, and biodiversity conservation.

As co-editors, we have enjoyed working with all authors and we sincerely thank them all for submitting their interesting work to this Research Topic.

With more exciting discoveries awaited for the future, we envisage that this Research Topic will serve as a foundation to stimulate further interest in gaining insights from evolutionary, cellular, eco-physiological and biochemical perspectives on invertebrates able to cope with extreme environments.

We hope our readers will appreciate this work, as much as we enjoyed putting it together.
